# Homeostatic regulation of spontaneous and evoked synaptic transmission in two steps

**DOI:** 10.1186/1756-6606-6-38

**Published:** 2013-08-22

**Authors:** Richard C Gerkin, David W Nauen, Fang Xu, Guo-Qiang Bi

**Affiliations:** 1 Department of Neurobiology, University of Pittsburgh, 15213 Pittsburgh, PA, USA; 2 School of Life Sciences, Arizona State University, 85287 Tempe, AZ, USA; 3 Center for the Neural Basis of Cognition, Carnegie Mellon University, 15213 Pittsburgh, PA, USA; 4 CAS Key Laboratory of Brain Function and Disease, and School of Life Sciences, University of Science and Technology of China, Hefei, Anhui 230026, China

**Keywords:** Homeostasis, Metaplasticity, Quantal hypothesis

## Abstract

**Background:**

During development both Hebbian and homeostatic mechanisms regulate synaptic efficacy, usually working in opposite directions in response to neuronal activity. Homeostatic plasticity has often been investigated by assaying changes in spontaneous synaptic transmission resulting from chronic circuit inactivation. However, effects of inactivation on evoked transmission have been less frequently reported. Importantly, contributions from the effects of circuit *inactivation* and *reactivation* on synaptic efficacy have not been individuated.

**Results:**

Here we show for developing hippocampal neurons in primary culture that chronic inactivation with TTX results in increased mean amplitude of miniature synaptic currents (mEPSCs), but not evoked synaptic currents (eEPSCs). However, changes in quantal properties of transmission, partially reflected in mEPSCs, accurately predicted higher-order statistical properties of eEPSCs. The classical prediction of homeostasis – increased strength of evoked transmission – was realized after explicit circuit reactivation, in the form of cells’ pairwise connection probability. In contrast, distributions of eEPSC amplitudes for control and inactivated-then-reactivated groups matched throughout.

**Conclusions:**

Homeostatic up-regulation of evoked synaptic transmission in developing hippocampal neurons in primary culture requires both the inactivation and reactivation stages, leading to a net increase in functional circuit connectivity.

## Background

Neuronal circuits require homeostatic mechanisms that modify synaptic weights in inverse relation to activity levels [1]. One such mechanism is synaptic scaling of excitatory synapses, in which mEPSCs are scaled up or down to counteract changes in neuronal activity [2-4]. In principle, changes in properties of mEPSCs reflect alterations in synaptic structure and signaling, and thus functional connectivity of neural circuits. However, the properties and machinery of spontaneous and evoked release could be quite distinct [5] (but see [6]). In any case, evoked “release probability” has no direct analogue in spontaneous neurotransmission. Thus it is unclear whether and how differences in mEPSCs can be directly mapped onto specific predictions of effects on evoked transmission (eEPSCs) and circuit function.

Besides synaptic scaling, another well-known mechanism of synaptic homeostasis is metaplasticity, where a reduction of neuronal activity can cause a homeostatic increase in the capacity for synaptic potentiation [7]. For example, after chronic inactivation followed by the return of activity, both synaptic scaling and increased capacity for Hebbian plasticity could contribute to long-term increases in synaptic strength and circuit connectivity [8,9]. However, it is not clear how scaling and metaplasticity each contribute to the total change in synaptic efficacy. Because they can occur simultaneously, and can even share molecular mechanisms [10], it has been difficult to distinguish their respective contributions.

## Results

### Mixed effects of chronic inactivation

To evaluate the effects of network inactivity on synaptic connectivity, we chronically blocked action potentials (APs) in developing hippocampal cultures with TTX. 24-96 hours later, we obtained perforated patch clamp recordings from pairs of nearby neurons in TTX-treated (n = 70) and control sister cultures (n = 77) (Additional file 1: Figure S1).

**Figure 1:**
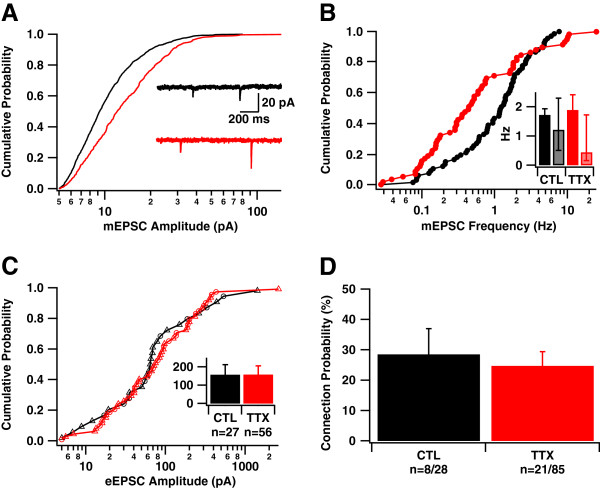
**Synaptic scaling after inactivation alone is reflected in the amplitude of miniature, but not evoked, currents. *A***, Following inactivation alone, mEPSC amplitude is enhanced in chronically TTX-treated culture; (p < 0.0001, KS test; n{CTL,TTX} = 1595,1390 events). Control cultures are shown in black, and TTX-treated cultures are shown in red. Inset: example mEPSCs. ***B***, The frequency of mEPSCs is differently distributed in TTX-treated culture (one point per cell; KS Test: p < 0.005; Bartlett’s test for equal variances: p < 10^-10^; n{CTL,TTX} = 68,60 cells); Inset: Mean +/- SEM (dark bars); median and interquartile range (light bars). ***C***, In paired recordings between random neurons, monosynaptic connections probed by the first stimulus following TTX washout showed evoked EPSCs (eEPSCs) of similar amplitude in control and TTX-treated networks. Inset: mean amplitude (p > 0.35, Wilcoxon Rank Test; p > 0.6, KS Test); circles indicate experiments conducted in 500 nM CNQX and renormalized to full amplitude; triangles indicate no drug. ***D***, The fraction of postsynaptic neurons showing an eEPSC in response to the first presynaptic stimulus was similar in control and TTX-treated cultures (p > 0.3).

Following such inactivation, we observed larger amplitude mEPSCs in TTX-treated cultures compared with control sister cultures (Figure 1A; CTL: 11.6 ± 0.2 pA; TTX 15.6 ± 0.5 pA; p < 0.0001), consistent with positive synaptic scaling [2,3,10,11]. The differences in mEPSC frequency were more complex (Figure 1B), reflected primarily by a lower median and broader distribution across cells in TTX-treated cultures (p < 0.005 Kolmogorov-Smirnov test, p < 10^-10^ Bartlett’s test for equal variances; CTL: 1.31 ± 0.45 Hz, median = 1.16 Hz; TTX: 1.53 ± 0.45 Hz, median = 0.46 Hz, ANOVA p > 0.7).

We next examined how these differences might be reflected in the magnitude of evoked synaptic currents (eEPSCs). We removed TTX from the bath medium only after establishing dual recordings, waited 20 minutes to ensure wash-out (Methods), and then applied single suprathreshold stimuli to one neuron while measuring a postsynaptic response in the other. Only experiments in which no spontaneous network activity was recorded prior to the first stimulus were used (67/72 recordings), avoiding the confound (see below) of activity-induced plasticity during the washout period.

To our surprise, neither the mean magnitude nor distribution of post-inactivation monosynaptic eEPSCs at excitatory connections between pairs of neurons in previously inactivated cultures was significantly different from those in control networks (Figure 1C; CTL: 157.6 ± 55.1 pA, n = 27; TTX: 158.3 ± 47.2 pA, n = 56; p > 0.3), nor was the connection probability, i.e. the probability of observing such a monosynaptic response at a putative connection (Figure 1D; CTL: 28.5 ± 8.5%; TTX: 24.7 ± 4.7%; p > 0.3). These results indicated that the differences in quantal amplitude and frequency, evident at the level of mEPSCs, had no direct correlates at the level of evoked synaptic transmission between cell pairs. However, we did observe that autaptic eEPSCs were weaker in controls than in inactivated networks (Additional file 1: Figure S2). Overall, synaptic scaling of quantal amplitudes did not guarantee an increase in the strength of evoked transmission in developing hippocampal neurons in primary culture.

### Contribution of quantal parameters to evoked transmission

The incongruity of effects of chronic inactivation on spontaneous and evoked transmission might have been due in part to differences in vesicle release probability *p*, which can be subject to homeostatic increase [12,13], since no obvious analogue to *p* exists in spontaneous neurotransmission. To determine whether putative changes in quantal size *q* and the number of synaptic boutons *n* could explain our results, with or without an increase in *p*, we probed the quantal hypothesis, which states that eEPSC amplitudes are equal to the product of *n*, *p*, and *q*[14], and which has been a useful tool for cataloging and identifying potential loci of synaptic plasticity [15]. Assuming mean mEPSC amplitude is proportional to *q*[16], and mEPSC frequency is proportional to *n*[17], we are still left with uncertainty about *p* (Additional file 1: Figure S3). To avoid this problem, we restated the quantal hypothesis to yield expressions independent of *q* and *n,* respectively (Methods). These are, respectively, the coefficient of variation (CV) and the Fano factor (FF) of eEPSC amplitude across trials, and these reflect the relationship between the variability and mean of these responses.

**Figure 2:**
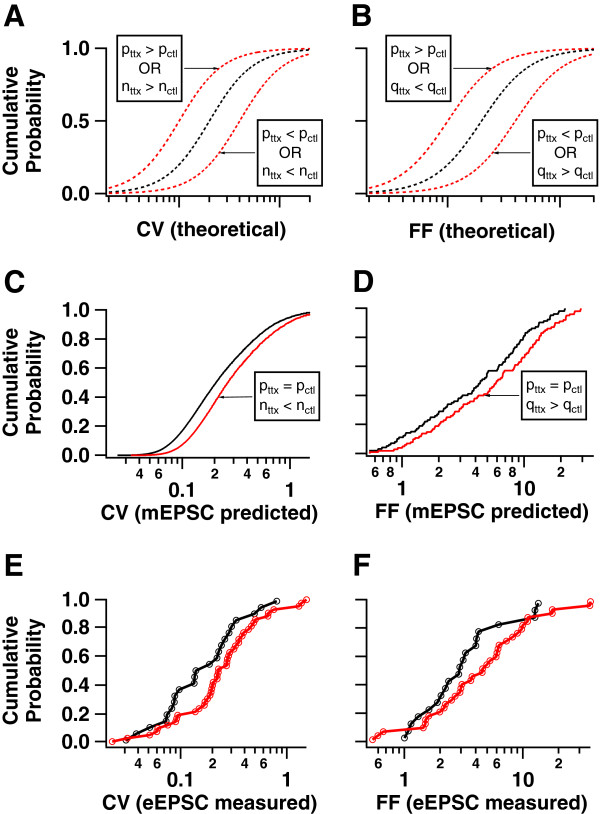
**Changes in eEPSCs are predicted by changes in mEPSC properties. *A***, ***B***, Hypothetical distributions of two measures of variability of evoked synaptic responses, the coefficient of variation (CV) and the Fano factor (FF), are illustrated with black lines. The red lines illustrate shifts in these distributions as a result of changes in quantal parameters *p*, *q*, or *n* indicated in the boxes. ***C***, Proxy distributions for the CV of an evoked synaptic response, a measure independent of quantal amplitude *q*. Distributions are generated using unitary synaptic connections built from experimental measurements of quantal parameters at single boutons (Nauen and Bi, 2012), along with changes in mEPSC properties measured in Figure 1. ***D***, Same as ***C***, but for the FF, a measure independent of bouton number *n*. ***E***, Measured distribution of the CV, taken from repeated measurements of eEPSCs recorded in pairs of neurons here. Each plotted point is obtained from the responses at one unitary connection. ***F***, Same as ***E***, but for the FF. Distributions in ***E*** and ***F*** are not significantly different from those predicted in ***C*** and ***D***, respectively (p > 0.2, KS test).

These quantities allow two null hypotheses about differences in quantal parameters at control (*ctl*) and chronically inactivated (*ttx*) synapses to be tested: (1) that the quantal amplitude composing eEPSCs changes in proportion to mEPSC amplitude changes (*p*_*ttx*_*≥ p*_*ctl*_, *q*_*ttx*_*/q*_*ctl*_*= ampl*_*ttx*_/*ampl*_*ctl*_); and (2) that the number of quanta composing eEPSCs changes in proportion to mEPSC frequency changes (*p*_*ttx*_*≥ p*_*ctl*_; *n*_*ttx*_*/n*_*ctl*_*= freq*_*ttx*_/*freq*_*ctl*_). Here, *ampl* and *freq* are measured properties of recorded mEPSCs, but {*p*,*q*,*n*} are estimated properties of evoked transmission. Qualitatively, each null hypothesis states that differences in mEPSC properties predict differences in quantal parameters of eEPSCs, given that the accompanying evoked release probability did not decrease as a result of inactivation. We assumed a non-decreasing *p* in these hypotheses because independent, direct measurements indicate that inactivation increases *p*[12,13]. Either or both of these hypotheses could be accepted or rejected by data, because these hypotheses make clear predictions about differences in CV and FF distributions resulting from inactivation (Figure 2A,B). Across conditions, observations of CV or FF sufficient to reject either null hypothesis would indicate that differences in mEPSC properties do not imply corresponding differences in quantal parameters of eEPSCs. Thus, the widely-assumed implication of synaptic scaling -- that it matters for evoked synaptic currents -- is directly tested by examining these measures.

To conduct this test, we computed proxy CV and FF distributions (Figure 2C,D) based on eEPSCs recorded from putative single boutons (Additional file 1: Table S1) [18] (under the lower bound (*p*_*ttx*_ = *p*_*ctl*_ ) of the null hypothesis (*p*_*ttx*_*≥ p*_*ctl*_); these proxy distributions were completely constrained by data (Methods). They specify the minimum magnitude of the rightward shift in both the CV (Figure 2A,C) and FF (Figure 2B,D) distributions.

If the rightward shift following inactivation in either the CV or the FF of eEPSCs was significantly smaller than the corresponding shift predicted in the proxy distributions, this would serve to reject the hypothesis that observed differences in mEPSC properties explain observed differences in eEPSC properties. However, the evoked distributions (Figure 2E,F) and their shifts were remarkably similar to the proxy distributions (CTL and TTX values were significantly different from each other, p < 0.05; but each was consistent with a proxy generated from its own quantal parameters in Figure 2C,D, p > 0.2). Thus the hypothesized decrease in *n* and increase in *q* suggested from mEPSC recordings is consistent with variability across trials observed in eEPSCs. This indicates that multiplicative scaling of both *n* and *q* is sufficient to explain changes observed in both spontaneous and evoked modes of transmission.

### Activity awakens latent changes in functional connectivity of previously inactivated networks

While examining eEPSCs in previously inactivated cultures, we observed that neuronal stimulation, especially that consisting of multiple pulses, could cause recurrent network activation as seen previously in comparable macro-island cultures [19]. Following such a protocol to achieve network *reactivation*, we again probed pairwise connectivity with single stimuli, followed by re-application of TTX to once again examine mEPSC properties.

**Figure 3:**
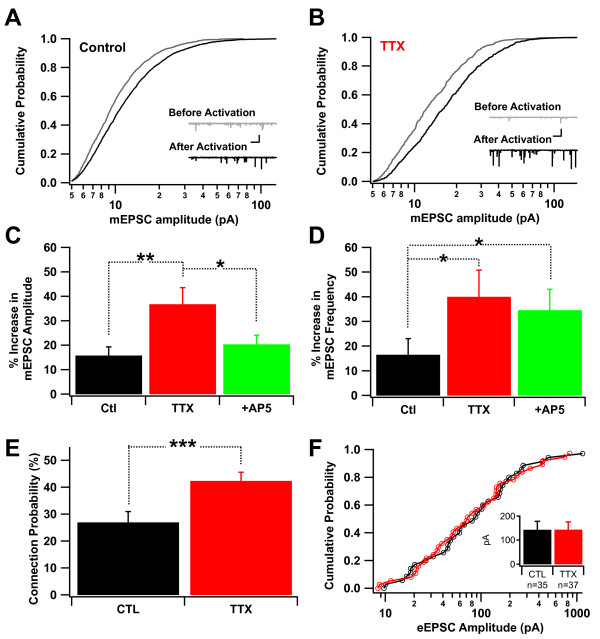
**Synaptic potentiation and connectivity are enhanced following reactivation. *A***, Cumulative histogram indicating mEPSCs recorded during 5 minute periods immediately before (gray) and 30 minutes after (black) a period of recurrent activation in control cultures (n = 16 cultures, one cell per culture). Insets show samples of the membrane current records (scale bars: 1 s, 25 pA). ***B***, Same as ***A***, but for TTX-treated networks (n = 7 cultures, one cell per culture). Thus, gray follows inactivation alone and black follows reactivation. ***C***, Summary data for the increase in mEPSC amplitude after (re)activation. ‶ + AP5″ in category label indicates acute application of AP5 during washout of TTX. ***D***, Similar to ***C***, except showing the increase in mEPSC log-frequency, illustrating that the potentiation is reflected as in increase in both the amplitude and frequency of mEPSCs. ***E***, Following (re)activation, an eEPSC is more probable in TTX-treated cultures, but ***F***, The amplitudes of these responses are similar to those of controls. * indicates p < 0.05; ** indicates p < 0.01; *** indicates p < 0.001.

We found that both mEPSC amplitude and frequency were increased 30 minutes after reactivation, compared with their values prior to reactivation (Figure 3A,B). Furthermore, these increases were greater in TTX-treated cultures than in controls subjected to the same activation protocol (Figure 3C, mEPSC amplitude: CTL: 115.8 ± 3.5%, n = 16 neurons; TTX: 136.8 ± 6.7%, n = 7; p < 0.01; Figure 3D, mEPSC log-frequency: CTL: 116.5 ± 6.5%; TTX: 140.0 ± 10.8%; p < 0.05). Differential levels of activity during reactivation did not explain this increase (vs. total charge, r^2^ < 0.1; vs. # of events, r^2^ < 0.1).

To determine the mechanistic similarity of reactivation-induced potentiation to NMDA receptor (NMDAR)-dependent long-term potentiation (LTP), we acutely subjected TTX-treated cultures to AP5 during reactivation. Despite the persistence of stimulus-induced recurrent network activation in the face of NMDAR block [19], significant reductions in the amplitude (but not frequency) component of the resultant reactivation-driven potentiation were observed (Figure 3C, mEPSC amplitude: TTX-treated + AP5: 120.4 ± 3.7%, n = 7; p < 0.05 vs. TTX-treated alone; Figure 3D, mEPSC frequency (log): TTX-treated + AP5: 134.6 ± 8.4%; p > 0.3 vs. TTX-treated alone), indicating that potentiation of mEPSC amplitude but not frequency during reactivation was partly NMDAR-dependent.

5-10 minutes after the onset of reactivation and initial measurement of pairwise connectivity, but immediately prior to re-addition of TTX, pairwise connectivity was tested again with single stimuli, to further examine the functional impact of reactivation. In contrast to observations made after withdrawal of inactivation alone (TTX washout) but prior to deliberate reactivation (Figure 1D), *after* such reactivation a larger fraction of potential excitatory connections between neurons was realized in TTX-treated cultures (Figure 3E; CTL: 27.0 ± 4.0%, n = 121; TTX: 42.4 ± 4.5%, n = 79; p < 0.005). Intriguingly, both the mean strength and the overall distribution of evoked excitatory responses were still very similar (Figure 3F; CTL: 143.7 ± 35.3 pA, n = 35; TTX: 144.1 ± 31.6 pA, n = 37; p > 0.5 by Wilcoxon Rank test). Consistent with a rapid enhancement of the connection probability in TTX-treated cultures by reactivation after TTX withdrawal, we observed the appearance of new monosynaptic connections between the randomly recorded neurons after reactivation (5/37 cases), but not after a similar activation protocol in controls.

## Discussion

Homeostatic synaptic regulation is crucial for stability in neuronal circuits [1,4]. Synaptic scaling is a result of such regulation, measured by differences in miniature synaptic currents; its impact on AP driven transmission has also been reported [3,20,21], but the relationship between these two manifestations of plasticity has been less investigated. We found that in developing hippocampal neurons, synaptic scaling of mEPSCs induced by chronic inactivity did not directly translate into enhancement in the mean amplitude of eEPSCs. Yet differences in quantal parameters inferred from mEPSCs are excellent predictors of higher-order statistics of evoked transmission, indicating the scope of co-regulation between spontaneous and evoked transmission. However, reactivation following inactivation is required to realize modification of a network’s functional connectivity.

### Effects of developmental context

Increased variance of mEPSC frequency after TTX treatment may be due to individual synapses adapting heterogeneously [22], or “drifting” away from equilibrium sizes [23], in response to inactivity. Overall, effects on bouton number, mEPSC frequency, and release probability are not consistent across experimental preparations, brain areas, or developmental stages [3,10,12,24-27]. In addition, our observation that inactivation alone had no appreciable effect on eEPSC size in developing hippocampal neurons (Figure 1) differs from reported scaling of eEPSC in cortical neurons [3,20], suggesting the variability of underlying mechanisms. A single molecular mechanism can even mediate opposing changes in mEPSC amplitude and frequency [28]. Since individual differences are so context-dependent, we focused not on their magnitude but on the correspondence – within the same system – between observed differences in mEPSCs and eEPSCs, in order to relate spontaneous to evoked synaptic transmission.

Cell-pair specific Hebbian modifications may give rise to network architectures constrained by topological rules of synaptic strength. For example, under normal conditions, autaptic inputs are weak compared to heterosynaptic inputs [29]. This principle may emerge from spike-timing-dependent plasticity (STDP), which predicts that autapses are weakened as a consequence of spiking: back-propagating APs precede autaptic currents in infiltrating dendritic segments [30], and repeated application of the STDP learning “rule” [31] then consistently depresses the magnitude of such currents. We hypothesize that in TTX-treated neurons, the absence of APs prevent this topological rule from emerging, because each neuron is unable to exploit AP timing information that identifies a synaptic input as coming from a cell’s own axon. Non-inactivated (control) neurons would have no such limitation, and their autapses might be weakened using this timing information. Our observation of relatively stronger autaptic eEPSCs in inactivated networks (Additional file 1: Figure S2) supports this hypothesis.

In this study, we have used primary hippocampal culture as a model system to study synaptic development and plasticity. While primary neuronal cultures have been widely used in many seminal experiments in the field, including the study of homeostatic plasticity and synaptic scaling [2,3,8,24], one must be careful when translating such *in vitro* results to interpretations of what happens in the intact brain. On the other hand, as the most fundamental biology is preserved, it is likely that mechanisms found in the reduced culture system could still manifest themselves *in vivo*, at least under certain developmental or modulatory conditions. As *in vivo* homeostasis in the hippocampus has been reported [32,33], it will be of interest for further studies to determine whether and how the two-step process observed here is operant in the intact brain.

### Effects of inactivation and reactivation

In our experiments, inactivity alone (Figure 1) had limited functional impact, since both eEPSC amplitude and connection probability were unaffected. What then is the function of homeostasis in this developing system? Figure 3 resolves this question: *reactivation* realizes the full homeostatic response. It further highlights the distinction between *inactivation* and *reactivation* that has been unappreciated because, in most preparations, neurons do not remain inactivated continuously prior to explicit reactivation. Because networks can become hyperexcitable following inactivation [34], it is difficult to enforce this control in many preparations; TTX washout alone can result in activity before deliberate reactivation, potentiating synapses [8]. However, we avoided spontaneous spiking prior to reactivation by (1) transferring and patching neurons before wash-out of TTX and (2) using island cultures with small networks of neurons (50-100), in which spontaneous activity is rare. Thus, in our preparation the effects of *inactivation* and *reactivation* on synaptic modifications have been temporally and mechanistically distinguished. While the potentiation of spontaneous synaptic currents by patterned activity has been shown previously, e.g. [35,36], the present results indicates that such potentiation is enhanced following inactivation, possibly due to the presence of new silent synapses [9].

Another possibility is a rapid increase in release probability following de-inactivation [37], which would complement increases in *q*. This latter possibility does not require postsynaptic spiking (but does require synaptic activation), and could thus occur even during the inactivation period to the extent that it can be activated by spontaneous transmission [38,39]. The increased connection probability could thus reflect a decrease in presynaptically silent synapses, a decrease in postsynaptically silent synapses, or the creation of entirely new synapses. Investigation of individual synapses with tools specific to pre- or postsynaptic machinery could help adjudicate among these possibilities.

### Differences between spontaneous and evoked release

We used a supranormal [40] extracellular calcium concentration (3 mM), which worked to our advantage by reducing cell excitability, thus preventing spontaneous reactivation after TTX withdrawal and allowing us to reactivate deliberately. At this concentration release probability is not saturated, however it does begin to depart from a simple power law concentration-dependence [41]. While there is no evidence that chronic inactivity and changes in extracellular calcium concentration increase release probability by the same mechanism, if they did then ceiling effects might reduce the observed increases in eEPSC size, relative to experiments with more modest extracellular calcium concentrations. In principle mEPSC statistics should be much less affected by calcium concentration. However, as shown in Figure 2, there is in no incongruity between changes in the statistics of mEPSCs and eEPSCs after inactivation, and that the apparent incongruity in Figure 1 comes from failing to consider release probability with equanimity across modes of release in the first place.

There has been debate about whether the machinery of spontaneous and evoked release are shared [6] or distinct [5]. If they are shared it should come as no surprise that the phenomenon of spontaneous and evoked release are co-regulated by inactivation and reactivation. In contrast if they are distinct then it is somewhat remarkable if they are so co-regulated. Given the sensitivity of our techniques, we cannot rule out quantitative differences in the changes to quantal parameters associated with each mode of transmission. Instead we show more modestly that a lack of quantitative differences is consistent with the data we collected (Figure 2).

### The regulatory target of synaptic homeostasis

The combined effect of inactivation and reactivation was an increase in connection probability and not on the mean or distribution of non-zero eEPSC amplitudes. How can this be, given observed potentiation of existing synapses? If new synapses are created/awakened, the amplitude distribution need not differ at all; as non-zero values reflecting existing functional connections increase in amplitude, new, small non-zero eEPSC amplitudes could be added to fill the “gap” in the distribution. Thus, while homeostatic synaptic adaptations may be intended to regulate firing rate output, they may also be subject to constraints that preserve, as in our data, the distribution of synaptic weights. In this view, while many statistics of synaptic transmission may be modified by plasticity in the short-run, connection probability may be a principal variable controlled by homeostasis in the long run. Indeed, the target(s) and timescale(s) of homeostasis may have been oversimplified in previous literature and deserve further attention in future studies [42].

## Conclusions and outlook

We have shown in primary cultured neurons that synaptic scaling of mEPSCs after inactivation does not guarantee increased eEPSC amplitude relative to controls. However, differences in quantal parameters inferred from mEPSCs predict measures of variability of evoked transmission, demonstrating that spontaneous and evoked transmission are nonetheless co-regulated. After reactivation (following inactivation) a more traditional measure of a network’s functional connectivity, the probability of a synaptic connection between pairs of cells, is elevated. But surprisingly the amplitude distribution of these functional connections is invariant to both inactivation and reactivation.

At the functional level, both scaling and metaplasticity can help restore the equilibrium of circuit activity in the face of small perturbations away from a homeostatic target. However, when a circuit is pushed far from equilibrium, like in the case of pharmacological silencing, or in stroke or traumatic brain injury, both scaling and metaplasticity may operate with magnitude and timescale ill-suited to functional recovery. They may overcompensate, producing circuits that are “too strong”, “too dense” and “too potentiatable” when they come online again. This in turn may lead to runaway activation associated with seizure [34,43].

## Methods

### Cell culture and pharmacology

Dissociated embryonic rat hippocampal neuron cultures were prepared as previously [44]. Cells were plated onto glass coverslips in 35-mm petri dishes at a density of 90000/dish. Coverslips were coated with poly-L-lysine (PLL) using a rubber stamp to form 1-mm diameter non-contiguous “islands” with 1.5 mm between island centers; each island usually contained 50-100 neurons. At 8–10 days *in vitro* (DIV), 0.5-1 μM tetrodotoxin (TTX) or vehicle was added to cultured neurons. Cultures were used for (interleaved) recording between 10–14 DIV, when recurrent activity is observed following stimulation [19]. Effects of inactivation duration were unremarkable, with scaling of mEPSC amplitude complete after 1 DIV in TTX, as reported elsewhere [21].

Perfusion was constant at ~1 ml/min during patch recordings. In recordings from TTX-treated cultures, TTX was present continuously during and after incubation and was removed only after recording began. Prior to removal of TTX, mEPSC amplitude and frequency were stable once steady-state membrane perforation by amphotericin B was reached. Acute addition of AP5 for TTX-treated cultures was concurrent with TTX washout. Complete TTX washout was confirmed by a plateau of Na^+^ mediated action current amplitude (Supplemental Methods). See Additional file 1: Figure S1 for further detail concerning the experimental timeline.

### Electrophysiology

Randomly selected pairs of neurons (50-150 microns apart) were recorded using perforated patch-clamp at room temperature. Presynaptic stimulation was given by step depolarization (100 mV, 1-2 ms), either single or paired (100 ms IPI) delivered to a single neuron at 30 s intervals. Initial stimuli to record eEPSCs did not begin until 20 minutes after a seal was obtained on the postsynaptic neuron, to allow for perforation of the membrane by amphotericin B. Final stimuli for recording EPSCs occurred 5-10 minutes later. In the intervening period, network activity and synaptic connectivity were monitored via patch recordings of 2-3 neurons in voltage clamp. Only monosynaptic connections, defined as producing an evoked EPSC (eEPSC) with a rising phase beginning less than 5 ms after the onset of the presynaptic stimulus, were considered in eEPSC analysis [45]. Control and inactivated eEPSC distributions were similar even when measured in 500 nM CNQX, ruling out contamination by polysynaptic eEPSC components (Additional file 1: Figure S4). Spontaneous mEPSCs were recorded in 0.5-1 μM TTX and 5 μM bicucculine methiodide (BMI). When BMI was absent, candidate event rates were largely unchanged, reflecting an extremely low rate of mIPSCs. Because spontaneous APs were rare, candidate event rates for control cultures were similar whether or not TTX was acutely applied prior to recording of mEPSCs (comparison within experiment, before and after acute TTX, p > 0.4, paired t-test). eEPSCs were identified only with the monosynaptic (short latency) component of an observed postsynaptic response.

### Quantal analysis

Evoked EPSCs (eESPCs) generated by quantal release with probability *p* and amplitude *q* at *n* potential synaptic boutons have a mean amplitude *npq*. The independence of these quantal parameters is evident both before and after chronic inactivation [46], and in single bouton data [18]. Thus, eEPSC variance is *n*<*q*^*2*^><*p*(1-*p*)>, where < > reflects a mean across boutons. Its coefficient of variation (CV_epsc_) and fano factor (FF_epsc_) are:

$$\begin{array}{l} CV_{\mathrm{epsc}}={\left(\mathrm{variance}\right)}^{1/2}/\mathrm{mean}=\left(1+CV_{q}{}^{2}\right)\\ \;\times \left(1-<p>\left(1+CV_{p}{}^{2}\right)\right)/{\left(n<p>\right)}^{1/2}\\ FF_{\mathrm{epsc}}=\mathrm{variance}/\mathrm{mean}=<q>\left(1+CV_{q}{}^{2}\right)\\ \;\times \left(1-<p>\left(1+CV_{p}{}^{2}\right)\right) \end{array}$$

where CV_x_ is the CV of quantal parameter *x*. Proxy total synaptic connections were generated by sampling (with replacement) *q* and *p* from putative single bouton recordings (Additional file 1: Table S1) [18]. Distributions of CV_epsc_ (CV) or FF_epsc_ (FF) under control conditions were computed according to the properties (<*p*>, <*q*>, CV_p_, and CV_q_) for each proxy. To generate proxy distributions for the inactivation condition, values of *q* and *n* at each bouton were multiplicatively scaled by differences in median mEPSC amplitude and frequency observed after chronic inactivation. CV_q_ and CV_p_ were left unchanged, consistent with pure multiplicative quantal scaling in TTX-treated cultures for the former, and lack of evidence either way for the latter. Thus, all proxies were derived from data. In each case, 1000 proxies were generated to construct the cumulative histograms. Discrete “steps” in the proxy distributions reflect finite combinations of *p* and *q* available in the single bouton data. “Measured” distributions for eEPSCs (Figure 2E,F) were obtained by measuring the mean and variance of eEPSCs across trials and computing CV and FF accordingly. Further details are provided in the Supplementary Experimental Procedures.

## Competing interests

The authors declare that they have no competing interests.

## Authors’ contributions

RCG co-designed the study, conducted most of the experiments, analyzed all the data, and wrote the manuscript. FX conducted some experiments presented in Figure 1 and in the supplemental materials. DWN contributed the single bouton data that was used in Figure 2 and Additional file 1: Table S1. GQB supervised the experiments, co-designed the study, and edited the manuscript. All authors read and approved the final manuscript.

## Supplementary Material

Additional file 1 **This file contains supplementary materials including supplementary experimental procedures, as well as the following figures and tables. Figure S1**: Experimental Timecourse. **Figure S2**: Autaptic currents are depressed by ongoing activity. **Figure S3**: Naïve prediction of evoked EPSC amplitude shift. **Figure S4**: Amplitude distribution of evoked synaptic currents measured in partial doses of CNQX is unchanged by chronic inactivation. **Table S1**: Single bouton measurements. **Supplementary Experimental Procedures**. Click here for file
