# Supplementary material for: Homeostatic regulation of spontaneous and evoked synaptic transmission in two steps
Source: Mol Brain. 2013 Aug 22;6:38. doi: 10.1186/1756-6606-6-38 (PMC3765453; doi:10.1186/1756-6606-6-38)
Supplement: Additional file 1 — This file contains supplementary materials including supplementary experimental procedures, as well as the following figures and tables. Figure S1: Experimental Timecourse. Figure S2: Autaptic currents are depressed by ongoing activity. Figure S3: Naïve prediction of evoked EPSC amplitude shift. Figure S4: Amplitude distribution of evoked synaptic currents measured in partial doses of CNQX is unchanged by chronic inactivation. Table S1: Single bouton measurements. Supplementary Experimental Procedures. [file 1756-6606-6-38-S1.docx]

**Figure S1: Experimental Timecourse.** The left column (black) and the right column (red) indicate the experimental time course for control and TTX-treated neurons, respectively.  Neurons are plated onto coverslips at E18 as described previously.  At 8-10 days in vitro, either TTX or vehicle is added to each dish of coverslips, with the former causing "inactivation".  After 1-4 more days, neurons are transferred from the incubator and patched, with TTX-treated neurons remaining in TTX throughout.  After perforation by amphotericin-B in the patch pipette, TTX is optionally washed-in acutely in controls (no differences were observed after acute TTX wash-in).  mEPSCs were recorded (no differences were observed in the presence of a GABA-A receptor antagonist, indicating an analysis template selective for mEPSCs over mIPSCs).  TTX was washed-out, thus "de-inactivating" neurons.  Suprathreshold stimulation was provided to single neurons, eliciting recurrent network activity, and causing "reactivation".  Monosynaptic eEPSCs were recorded between pairs of neurons during these stimuli.  TTX was washed in, and mEPSCs recorded once again following the period of reactivation.

**Figure S2: Autaptic currents are depressed by ongoing activity.  *A***, The contribution of AMPAR currents alone to an autaptic response is isolated by recording responses in CNQX (5 µM), revealing unambiguous depression of the AMPAR-mediated synaptic current following 60 suprathreshold intracellular current injections. ***B***, The fraction of cells exhibiting autaptic currents is similar in control and inactivated networks (n{control, inactivated} = 157, 259 cells). ***C,*** The amplitude of autaptic currents is substantially smaller in control networks than in inactivated networks. Inset shows mean across autapses (*** = p<0.0005).

**Figure S3:** **Naïve prediction of evoked EPSC amplitude shift.** Multiplying mean mEPSC amplitude (*q*) by mean mEPSC frequency (*n*) in each recorded cell produces a naïve estimate of evoked EPSC amplitude. This assumes that each evoked EPSC consists of *n* synaptic boutons releasing quantal content *q* with release probability 1.

**Figure S4: Amplitude distribution of evoked synaptic currents measured in partial doses of CNQX is unchanged by chronic inactivation.  *A***, Monosynaptic eEPSCs from connected pairs were subject to various concentrations of CNQX to create a dose-response curve. ***B***, At the culture density used for the experiments in the main text, there is no difference in the amplitudes of evoked (non-zero) synaptic connections measured under 500 nM CNQX between control and TTX-treated networks (p>0.5, Wilcoxon Rank Test). Amplitudes were normalized to a CNQX-free condition using the data from panel A.

**Table S1: Single bouton measurements.** Mean peak eEPSC release probabilitites (1 - failure rate) and amplitudes from putative single bouton recordings in 10 different pairs of neurons in hippocampal cultures. These eEPSCs were obtained from single boutons using a localized perfusion method during dual perforated-patch recordings (Nauen and Bi, 2012). Briefly, normocalcemic extracellular perfusion solution was applied in a laminar stream to a small region of the axon using focal positive and negative pressure while the global solution contained insufficient calcium for evoked release. These {release prob., amplitude} value pairs were sampled with replacement to create proxy synaptic connections between pairs of neurons for the analysis in Fig. 2 of the main text. The numbers of sample pairs used to create each connection (i.e. the number of single boutons per macrosynaptic connection) was chosen to be the mean of experimentally measured eEPSCs between pairs of neurons divided by the mean amplitude of single bouton eEPSCs from these 10 pairs.

**Supplementary Experimental Procedures**

**Electrophysiology**

The pipette solution contained (in mM): K-gluconate 136.5, KCl 17.5, NaCl 9, MgCl_2_ 1, HEPES 10, EGTA 0.2, and 200 µg/ml amphotericin B (pH 7.2). The external bath solution contained (in mM): NaCl 150, KCl 3, CaCl_2_ 3, MgCl_2_ 2, HEPES 10, and glucose 5 (pH 7.4). Recordings showing significant changes (>10%) in series (20-40 MΩ) or input resistance (500-1500 MΩ) were excluded.

For determining neurotransmitter phenotype, an eEPSC at any latency < 50 ms in any recorded postsynaptic partner was considered evidence that a presynaptic cell was excitatory (glutamatergic), since GABAergic neurons cannot evoke a polysynaptic response at this stage in development. To avoid the potential confound of polysynaptic responses in measuring the monosynaptic connection between neurons, a subset of experiments were also conducted in the presence of 500 nM CNQX to further isolate the monosynaptic component. After renormalizing the EPSC using dose-response curves (Fig. S4), the CNQX and non-CNQX data sets showed similar distributions of responses (p>0.4 by Kolmogorov-Smirnov Test), so these data were pooled for further analysis. IPSCs were distinguished from EPSCs by their much slower decay kinetics, and in some case verified by their reversal potential. Connections with monosynaptic IPSCs were excluded.

**Analysis and Statistics**

Candidate mEPSCs were identified using an established method (Kudoh and Taguchi, 2002), with a threshold of 5 pA (~3 times the standard deviation of the noise). The potentiation of mEPSC frequency varied greatly across recordings, in part due to variability in the initial frequency. In order to normalize comparison, we computed the logarithm of the multiplicative change in frequency before computing statistics.

Data collection, analysis, and production of figures were done using Igor Pro (Wavemetrics). Comparisons of binary data were done using a binomial test, and all other comparisons were made using a nonparametric Wilcoxon rank test (for skewed distributions), or an ANOVA with "TTX treatment" and "DIV recorded" as the covariates. A Bonferroni correction for multiple comparisons to the same control was made where applicable. Comparisons to unity (100%, no change in synaptic strength) remain uncorrected. Values are reported as mean ± SEM, and all error bars in graphs are SEM, unless indicated otherwise.

**TTX washout**

To confirm that TTX washout was complete at the time of first stimulation, in a separate set of experiments, periodic stimulation was delivered beginning immediately after washout was initiated, and the Na^+^-mediated action current reached steady state within ~5 minutes, indicating that TTX no longer blocked a measurable fraction of Na^+^ channels after this time point. In the experiments analyzed here, first stimulation was not delivered until >20 minutes after washout initiation, and we observed no change in the amplitude of action currents after this time point. Furthermore, residual un-washed TTX, which might be expected to decrease the effective *n* and/or *p*, is inconsistent with the direction of the observed differences in FF and CV in Fig. 2, and thus we rule out the possibility that slow TTX washout confounded our results. Spontaneous reactivation occurred rarely after washout in a subset of experiments, and such network activity sometimes occurred spontaneously in controls. We excluded those experiments from analysis because effects of inactivation and those of reactivation were confounded.
